# Reservoir parameters prediction based on spatially transferred long short-term memory network

**DOI:** 10.1371/journal.pone.0296506

**Published:** 2024-01-30

**Authors:** Wancheng Huang, Yuan Tian

**Affiliations:** Business School, Sichuan University, Chengdu, Sichuan, China; Sunway University, MALAYSIA

## Abstract

Reservoir reconstruction, where parameter prediction plays a key role, constitutes an extremely important part in oil and gas reservoir exploration. With the mature development of artificial intelligence, parameter prediction methods are gradually shifting from previous petrophysical models to deep learning models, which bring about obvious improvements in terms of accuracy and efficiency. However, it is difficult to achieve large amount of data acquisition required for deep learning due to the cost of detection, technical difficulties, and the limitations of complex geological parameters. To address the data shortage problem, a transfer learning prediction model based on long short-term memory neural networks has been proposed, and the model structure has been determined by parameter search and optimization methods in this paper. The proposed approach transfers knowledge from historical data to enhance new well prediction by sharing some parameters in the neural network structure. Moreover, the practicality and effectiveness of this method was tested by comparison based on two block datasets. The results showed that this method could significantly improve the prediction accuracy of the reservoir parameters in the event of data shortage.

## 1 Introduction

Logging curves provide a continuous record of various physical properties correlated with the depth of oil and gas wells. Common acquisition methods include nuclear magnetic resonance logging, acoustic logging, electric logging, among others. However, these methods are costly as they necessitate interruption of the drilling process for implementation. The collaborative examination of geological characteristics, petrophysical properties, and geophysical logging data pertaining to carbonate reservoirs proves effective in discerning intricate geological structures within the wellbore. This integrated approach contributes significantly to optimizing decision-making in oil and gas exploration and production [1]. While each piece of logging data is invaluable, instances of data loss may occur due to factors such as formation damage, well caving, and tool jamming. Consequently, a strategic approach is imperative to address data scarcity by leveraging one dataset to predict another [2]. This necessitates the development of robust methodologies to mitigate data loss and enhance the reliability of predictions.

For the efficient production of oil and gas fields, machine learning techniques have been applied to reservoir reconstruction to forecast important metrics like raw porosity and reservoir permeability, which can be attributed to the rapid growth of big data and artificial intelligence. A predictive model may be trained with a huge amount of historical data to map the link between geophysical logs and reservoir characteristics if sufficient geophysical and petrophysical understanding are not required.

Well logs have demonstrated their immense value within the petroleum industry, primarily for their pivotal role in assessing reservoirs [3]. Numerous studies on prediction techniques and accuracy have been conducted, and some significant advancements have been made. A Back-Propagation of Error Artificial Neural Network (BP-ANN) was created by Hamidi et al [4], which can be used to forecast the porosity of formations. The BP-ANN has been found to be more precise than Geolog Software (GS) in estimating oil reservoir porosity. To completely capture the useful information contained in log data and enhance the precision of porosity prediction, Wang et al [5] proposed a unique deep learning approach based on the one-dimensional convolutional neural network and bidirectional gated recurrent unit neural network. An optimized propagation RBF network was created by Baneshi et al [6], which could accurately predict the porosity index. To improve the shortcomings of individual neural networks in terms of porosity prediction, Duan et al [7] presented a new neural network model based on BP neural network, radial basis function (RBF) neural network, and support vector regression (SVR) mode. The long-short memory neural network (LSTM) was introduced by Zhang et al [8] into the logging curve synthesis, and the results of real logging data verification revealed that the synthesized logging curve of the LSTM neural network is more accurate than that of the fully connected neural network (FCNN), which more suitable for solving complex problems. A hybrid data-driven model consisting of a PSO, SVR, and deep learning network was built by Gu et al [9] to produce more accurate predictions. Reda Abdel Azim et al [10] propose an artificial neural network (ANN) model based on the back propagation learning algorithm to predict formation permeability from well logs, using a weight visualization curve technique to optimize the number of hidden neurons and layers. In terms of model design, structural modification, parameter setting, and data pre-processing, existing approaches for the prediction of reservoir parameters tend to deliver better results. Deep learning requires large amounts of data to be involved in training [11], however small datasets render them worthless for new reservoir blocks in the absence of previous data. New wells lack training data [12], and old historical data cannot be used directly to train the new well prediction model because of differences in geological conditions. In such scenario, there is an urgent need for a method that makes effective use of historical data and can eliminate spatial discrepancies.

To address data shortages, David et al [13] present a novel data-driven model based on a nonlinear autoregressive neural network with exogenous input to estimate shear and compressional sonic travel time, using petrophysical data as a substitute for acoustic measurements. The model offers a simple and robust approach for accurately estimating sonic logs, reducing the amount of required input data. Han et al [14] propose integrating deep neural networks (DNN) and ensemble learning machines (ELM) to accurately estimate missing well logs in the oil/gas industry. The proposed method addresses the limitations of traditional methods, such as multiple linear regressions and machine learning techniques, in capturing the complex and nonlinear relationships among well logs. Shan et al [15] addressed the issue of data scarcity in well log prediction by employing a hybrid neural network architecture that integrates BiLSTM (Bidirectional Long Short-Term Memory), an attention mechanism, and CNN (Convolutional Neural Network). This innovative approach takes into account the spatio-temporal information embedded in well logs, effectively mitigating the challenges associated with missing data. Wu et al [16] introduced a CNN-LSTM-PSO model that seamlessly combines the power of Convolutional Neural Network (CNN), Long Short-Term Memory Neural Network (LSTM), and Particle Swarm Optimization (PSO) algorithm. This model effectively and accurately forecasts well log data while taking into account spatio-temporal information, providing a resilient solution to tackle the challenge of data scarcity in well logging.

Most of the existing studies focus on the selection and parameter optimization of deep learning models, but the prediction power of these models is substantially limited when faced with the shortage of training data. No matter how good the models are in terms of feature extraction and how reasonable the model parameters are set, the inherent data shortage predefine the fact that the models cannot extract enough knowledge. To overcome this problem, some scholars have proposed prediction models based on gray system [17, 18], while others have adopted a virtual sample generation method to compensate for the data shortage [19]. However, these models based on virtual sample generation (VSG) or traditional machine learning models are less practical for predicting oil and gas reservoir parameters in real situations. Usually, a huge amount of historical data is accumulated during oil and gas reservoir exploration and development, and for the data shortage of newly explored blocks or unconventional reservoirs, application of transfer learning can make full use of the existing historical data.

In this paper, transfer learning is introduced into reservoir parameter prediction using deep learning networks. It makes full use of the common features of logging data from different blocks and solves the problem concerning prediction accuracy caused by insufficient data in small sample prediction. Transfer learning can extract common features from datasets that obey different data distributions. Few research has been done on applications using transfer learning in reservoir parameter prediction. In order to enhance the accuracy of neural network that records reservoir parameter prediction by correlating reservoir parameters, Shao et al [20] developed correlation-based transfer learning. The above findings demonstrate that transfer learning can considerably enhance the predictive power of both the permeability model and the water saturation model.

This study pioneers an exploration into the data shortage challenge within reservoir parameter prediction, addressing a critical gap in current research. Building on insights from various fields, such as traffic flow prediction [21], location-based services, and Point-of-Interest (POI) recommendation [22], as well as continuous-time sequential recommendation [23], we introduce a state-of-the-art transfer learning model based on the long short-term memory (LSTM) architecture. Moreover, our proposed model transcends the realms of academia by not only enriching scholarly discussions but also offering a pragmatic solution for refining reservoir parameter prediction in real-world settings. This dual-dimensional contribution lends credence to its significance in both theoretical and practical contexts.

Going beyond traditional methodologies, our model stands out in three fundamental dimensions:

(1) Model Innovation: Our transfer learning model, anchored in LSTM, introduces a novel approach to alleviating the data scarcity predicament in reservoir parameter prediction. Its superiority over conventional methods underscores its methodological innovation and advancement in predictive accuracy.
(2) Feature Extraction Ingenuity: Uniquely, our research demonstrates innovation in feature extraction by leveraging the LSTM underlying model. Given its aptitude for handling sequential data, such as well logging data resembling sequential patterns, this approach showcases a pioneering method for enhancing reservoir parameter prediction.
(3) Practical Applicability: Crucially, our research is not confined to academic discourse; it serves as a practical solution for real-world reservoir parameter prediction challenges. By offering tangible benefits to reservoir management and decision-making processes, our model provides a valuable tool for stakeholders in the field.

A case study was carried out in the two distinct blocks of Gaomo and Chuanxi to evaluate the proposed networks. To assess the transfer learning models’ performance in making predictions with the same dataset, we used three more models trained on the source only data, the target only data, the mixed data of source and target, but without transfer learning. The outcomes demonstrate that the suggested neural network may raise prediction accuracy through transfer learning. Additionally, several tests were presented to assess the method’s resilience and robustness, and the outcomes showed its broad application in forecasting reservoir characteristics in various blocks of wells.

## 2 Methodology and meterial

### 2.1 Transfer learning

**Fig 1:**
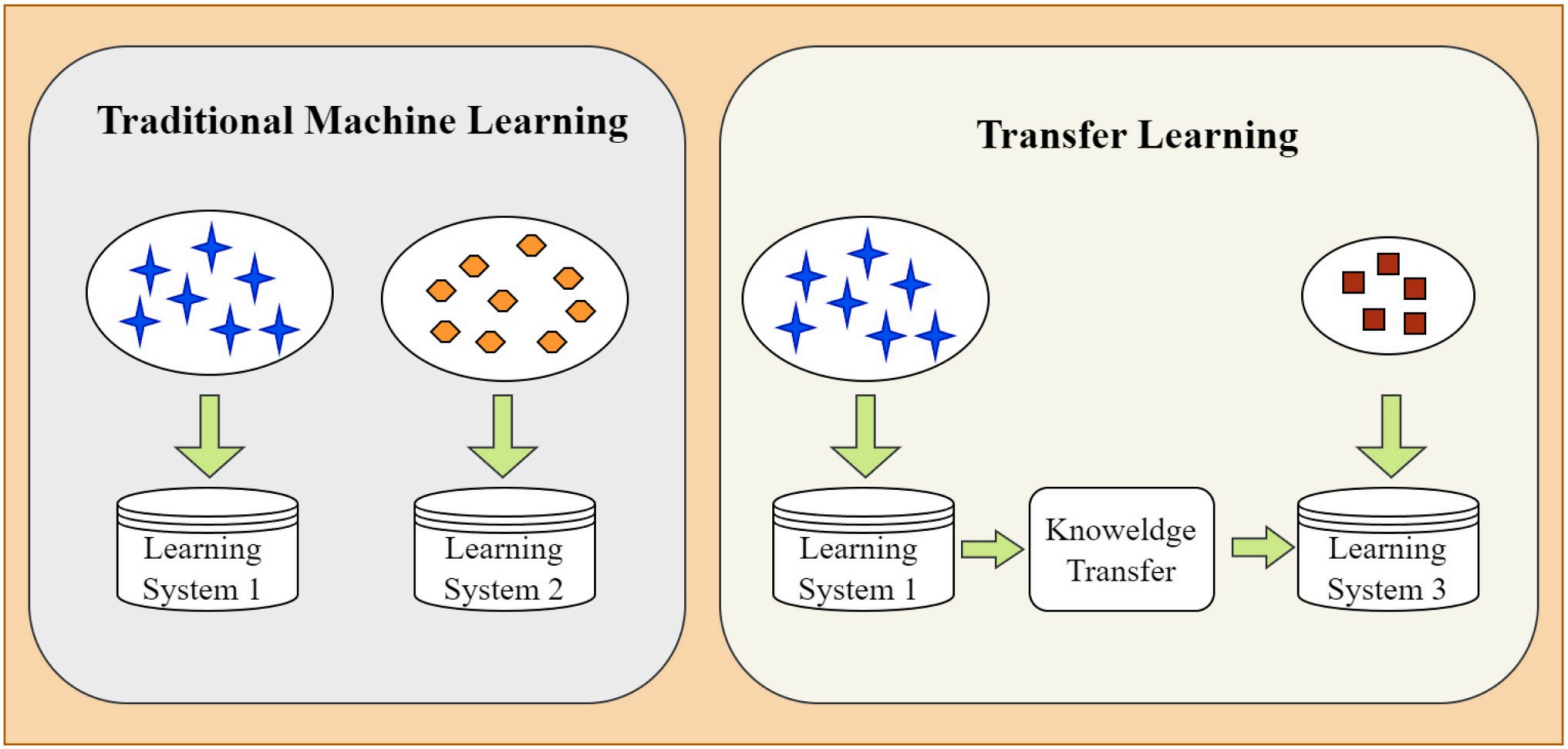
Learning process of transfer learning.

The correlation between previous and new learning activities can be utilized to learn new information and solve new challenges. In the realm of artificial intelligence, efforts are also being made to create a link between old and new knowledge, a process known as transfer learning. Transfer learning, in particular, uses data, tasks, or model similarities to apply models and information learnt in one domain to another. The proposed fundamental tenet of transfer learning is depicted in Fig 1.

Transfer learning is defined as follows:

$$\begin{array}{c} \left\{ \begin{array}{c} D=\{\chi ,P(X)\}\\ T=\{y,f(\cdot )\} \end{array}\right. \end{array}$$ (1)

Where *D* and *T* denote domain and task, *χ* and *y* are feature space and labels space, and *P*(*X*) and *f*(⋅) denote marginal probability distribution and target prediction function, respectively.

In brief, for a given source *D*_*S*_ domain and learning task *T*_*S*_, a target domain *D*_*T*_ and learning task *T*_*T*_, transfer learning can improve the prediction accuracy of the prediction function *f*_*T*_(⋅) on the target task *T*_*T*_ by effectively using the source domain *D*_*S*_ data to compensate for the lack of target domain *D*_*T*_ data. Based on the different types of focus, transfer learning can be divided into instance-based transfer, feature-based transfer, shared parameter-based transfer and relation-based transfer learning [24]. The instance-based transfer selects from the source domain instances that can be used for training in the target domain, such as effective weight distribution of labeled data instances. In this way, the distribution of instances between these two domains is close, which help to foster a reliable learning model with high classification accuracy in the target domain. However, the data distributions are inconsistent in these two domains in transfer learning, so all the labeled data instances in the source domain may not be useful for the target domain. The feature-based transfer maps the data in the source and target domains from the original feature space to a new feature space in which the data have the same distribution, allowing better use of the existing labeled data samples in the source domain for classification training and eventually for classification testing of the data in the target domain of the new space. The transfer based on shared parameters requires that each relevant model in the task has some shared and identical parameters or prior distributions, which can be further processed to achieve the transfer purpose. Relationship-based transfer learning which mainly mines and uses relationships for analogy transfer is rarely used. The main algorithms that are widely used in transfer learning are Domain-Adversarial Neural Network (DANN) [25], TrAdaboost [26]. DANN is a feature-based transfer learning method that includes feature extraction, classifier and domain discriminator. DANN adds an adversarial mechanism to the training so that the feature extractor and the domain discriminator are trained against each other as a way to learn features common to the source and target domains. TrAdaboost is an instance-based transfer learning method which achieves transfer by increasing the weight of instances that favor the target task while decreasing that of instances that do not favor the target task.

Transfer learning is a significant subfield in machine learning, and it has been applied to various industries. Transfer learning may be helpful for any application that suits the above scenario, which include, but are not restricted to, computer visual recognition [27], text categorization [28], activity recognition [29], natural language processing [30], human-computer interaction [31], and others. Table 1 summarizes the corresponding mainstream algorithms for several types of transfer learning methods.

**Table 1:** Summary of transfer learning method.

Category	Transfer learning method
Instance-based transfer	KMM [32], TrAdaBoost [26], WANN [33]
Model-based transfer	FineTuning [34], Knowledge Distilling [35], RegularTransfer [36]
Feature-based transfer	TCA [37], CORAL [38], DANN [25]
Relation-based transfer	Markov Logic Net [39]

### 2.2 Pretrain-finetune

**Fig 2:**
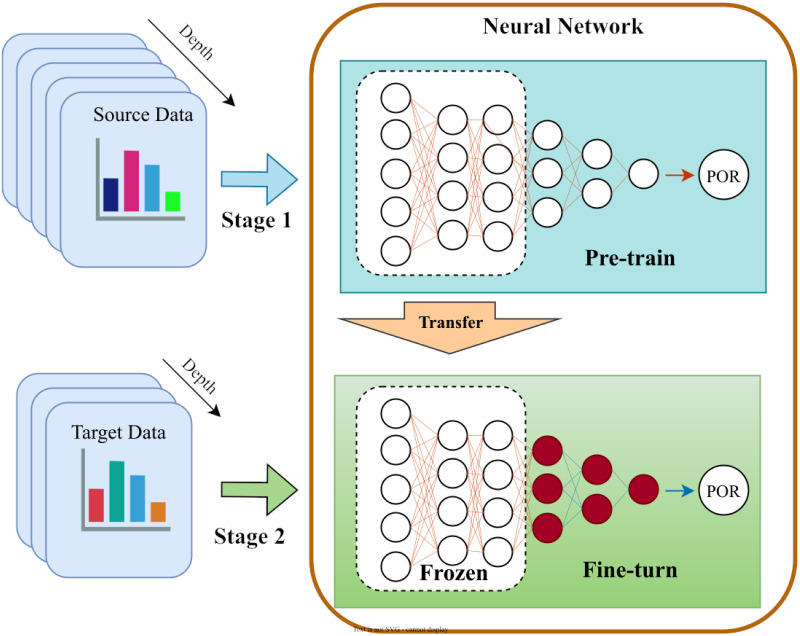
The working process of Pretrain-finetune.

The model-based transfer is a method to find the parameter information shared between the source and target domains in order to achieve transfer. This transfer approach is based on the assumption that the data in the source domain and those in the target domain can share some parameters of the model, with Pretrain-finetune being one of the most typical methods. Fig 2 shows a simple Pretrain-finetune process. For a task that requires transfer learning, we first train a model on the source domain, and then transform this network by fixing the parameters of the first few layers and fine-tuning the next few layers only for the task in the target domain. With some shared parameters in the model, a significant improvement in network training speed and prediction accuracy has been obtained.

### 2.3 Transfer learning based LSTM

LSTM (Long Short-Term Memory) is a special kind of recurrent neural network, whose core idea is to add the concept of gate to control the RNN units in the ordinary ones. It has improved the long-term dependency problem in traditional RNN. The gate mechanism includes three types: forget gates *f*_*t*_, input gate *I*_*t*_ and output gate *O*_*t*_.

The function of the forget gate *f*_*t*_ is to determine which information should be discarded or retained; that of the input gate *I*_*t*_ is to determine how much new information is added to memory cell *C*_*t*_; and that of output gate *O*_*t*_ to determine the output value. The memory cell *C*_*t*_ can update information under the joint action of forget gates *f*_*t*_, input gate *I*_*t*_ and output gate *O*_*t*_. The basic formula of the LSTM is as follows:

$$\begin{array}{c} \left\{ \begin{array}{c} f_{t}=\sigma (W_{f}\cdot [h_{t-1},x_{t}]+b_{f})\\ I_{t}=\sigma (W_{i}\cdot [h_{t-1},x_{t}]+b_{i})\\ {\tilde{C}}_{t}=\text{tanh}(W_{C}\cdot [h_{t-1},x_{t}]+b_{g})\\ C_{t}=f_{t}*C_{t-1}+I_{t}*{\tilde{C}}_{t}\\ O_{t}=\sigma (W_{O}\cdot [h_{t-1},x_{t}]+b_{O})\\ h_{t}=O_{t}*\text{tanh}(C_{t}) \end{array}\right. \end{array}$$ (2)

Where the weight matric *W*_*f*_, *W*_*i*_, *W*_*C*_, *W*_*O*_ and bias vector *b*_*f*_, *b*_*i*_, *b*_*C*_, *b*_*O*_ are obtained by the training. *σ* and tanh respectively represents the sigmoid and tanh activation function.

The gating mechanism introduced by LSTM plays a pivotal role in alleviating the issue of gradient vanishing [40]. In comparison, traditional RNN suffer from the problem of constant derivatives of previous memory to the next memory, leading to certain partial derivatives in RNNs always being either greater than 1 or within the [0, 1] range. This phenomenon results in the well-known problems of gradient vanishing or exploding. In contrast, LSTM effectively addresses this problem by dynamically adjusting its gating values, thereby maintaining the gradients within a reasonable range and facilitating more appropriate gradient propagation.

Furthermore, the gating mechanism in LSTM provides an invaluable feature filtering function. It allows LSTM to retain pertinent features while discarding extraneous ones, significantly enriching the information within vector representations. The distinctive chained neural network structure characterizes LSTM as a recurrent neural network, where each time step within the network selectively preserves, updates, and outputs information to subsequent time steps [41].

LSTM enjoys unparalleled advantages in processing time series data, while the relationship between logging data features and logging depth is similar to that of time series data. Chen [42] applied a multi-layer long and short-term memory(MLLSTM) model model to reservoir porosity prediction. LSTM was shown to have better robustness and accuracy than gated recurrent units (GRU) and recurrent neural networks (RNN) in depth sequence prediction. Therefore, LSTM was used as the underlying neural network structure for transfer learning in this study. Chen et al [43] have introduced an innovative Integrated Long Short-Term Memory (EnLSTM) network that strategically merges Integrated Neural Networks (ENNs) and Cascaded Long Short-Term Memory (C-LSTM) networks. This integration effectively harnesses their complementary strengths, offering a compelling solution to confront the intricate challenges associated with small data problems, notably addressing issues like over-convergence and training failures.

In this paper, the LSTM model is employed as the foundational framework, seamlessly integrated with a transfer learning approach to address the issue of data scarcity in the domain of logging data prediction. The method proposed in this study is the Transfer Learning-based LSTM(TL-LSTM), which effectively combines the advantages of deep learning and transfer learning to solve the problem of re servoir parameter prediction. Details of the proposed method are shown in the Algorithm 1.

**Algorithm 1 Pseudo code of the TL-LSTM algorithm**

1: Initialize an LSTM model

2: **for** i from 1 to *NS* **do**

3:  Calculate regression loss

4:  update network weight parameters

5: **end for**

6: Freeze the first few layers of the model

7: **for** i from 1 to *NT* **do**

8:  Calculate regression loss

9:  update network weight parameters

10: **end for**

11: **return** TL-LSTM

### 2.4 Data description and preprocessing

In this paper, data were derived from two major oil and gas well blocks in Sichuan Province, namely Gaomo and Chuanxi. As shown in the Table 2, the geological conditions in the west Sichuan block are more complex, making it more difficult to obtain the logging data, so there is an urgent need to use transfer learning to solve the problem of accuracy caused by the lack of training samples. We selected one well each from the Gaomo and Chuanxi blocks as the source and target wells, and named them Gaomo 001 and Chuanxi 001, respectively. After feature screening, we filtered out nine input variables with low missing rates and one target variable, i.e., porosity. Table 3 summarizes the parameters and abbreviations of the input and target variables used in this study.

**Table 2:** Geological characteristics of typical carbonate reservoirs in the Sichuan Basin.

Block	Gaomo	Chuanxi
Deepth	5000*m*-5500*m*	7100*m*-7500*m*
Formation temperature	153°C	160°C
Reservoir thickness	60*m*-150*m*	20*m*
Porosity	3.87%	3.11%
Permeability	0.51*mD*	0.27*mD*-1.73*mD*
Crustal stress	91*MPa*-96*MPa*	150*MPa*-160*MPa*
Reservoir type	Crack-hole type, hole type, pore type	Crack-hole type

**Table 3:** Parameters and abbreviation for input and target variables used in this study.

Parameter type	Features	Abbreviation
Input variables	Acoustic	AC
	Caliper Log	CAL
	Compensated Neutron Log	CNL
	Drift Azimuth	DAZ
	Density	DEN
	Deviation	DEV
	Gamma Ray	GR
	Photoelectric Effect	PE
	Water Saturation	SW
**Target variables**	**Porosity**	**POR**

Outlier data points were cleaned since the Logging data was automatically collected by sensors and artificially generated in part. The normalization method was used to de-quantize the data, and then 17 samples were randomly selected from the target dataset as the training set and the rest as the test set.

$$\begin{array}{c} z=\frac{x-\mu }{\sigma } \end{array}$$ (3)

Where *x* is the input variable, *μ* is the mean and *σ* is the standara deviation.

**Fig 3:**
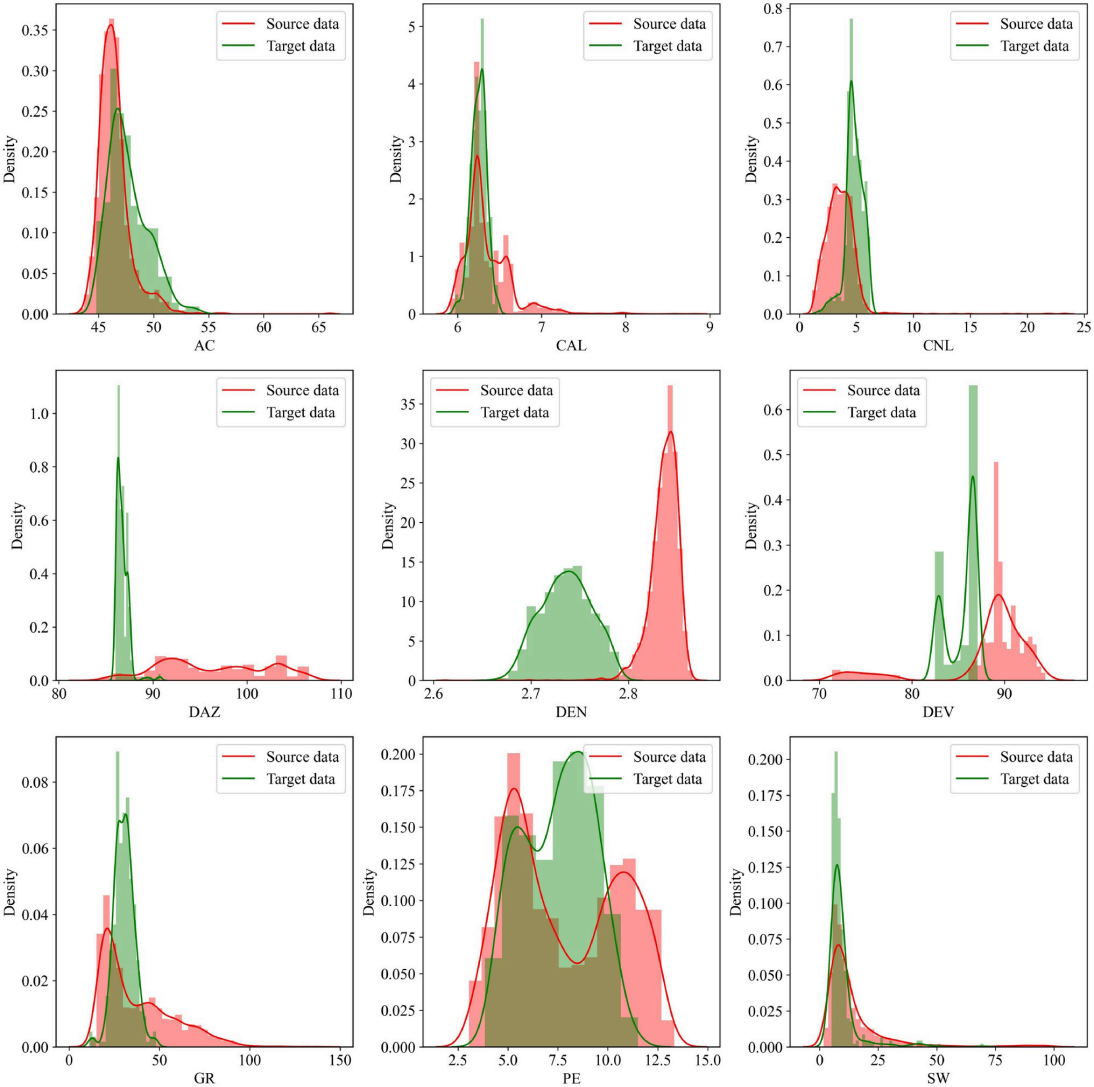
Feature distribution comparison of Gaomo 001 and Chuanxi 001.

Tables 4 and 5 and Fig 3 summarize the data distribution of the source and target datasets. It can be seen that the distribution of the source data and that of the target data are quite different. Among them, the data distributions of these two wells are closer on three features, i.e., AC, CAL, and SW. On the remaining six input features, the two distributions, however, are more different.

**Table 4:** Feature distribution description of source well dataset.

	AC	CAL	CNL	DAZ	DEN	DEV	GR	PE	SW	POR
**count**	3791	3791	3791	3791	3791	3791	3791	3791	3791	3791
**mean**	46.442	6.3443	3.5345	96.331	2.8362	88.159	37.473	7.7942	14.492	2.3207
**std**	1.5058	0.2756	1.3143	5.6753	0.0154	5.3124	19.807	2.8064	14.858	1.1332
**min**	43.226	5.91	1.117	84.407	2.612	71.351	11.649	3.043	1.727	0.032
**25%**	45.523	6.195	2.724	91.829	2.829	88.456	21.224	5.3145	6.914	1.497
**50%**	46.236	6.255	3.482	95.705	2.838	89.297	29.897	7.183	9.706	2.274
**75%**	46.985	6.472	4.284	101.19	2.846	90.937	49.663	10.469	15.313	3.051
**max**	66.016	8.803	23.299	106.55	2.871	94.401	138.25	13.319	99.77	6.742

**Table 5:** Feature distribution description of target well dataset.

	AC	CAL	CNL	DAZ	DEN	DEV	GR	PE	SW	POR
**count**	350	350	350	350	350	350	350	350	350	350
**mean**	47.775	6.2546	4.8426	86.749	2.7359	85.401	30.242	7.5944	9.2923	4.9718
**std**	1.7531	0.0919	0.7647	0.6543	0.0259	1.637	5.437	1.7557	6.168	1.2303
**min**	44.798	5.973	1.629	86.017	2.67	82.506	11.03	3.849	5.108	0.886
**25%**	46.517	6.1985	4.43	86.27	2.7182	83.37	26.92	6.039	6.491	4.2912
**50%**	47.449	6.265	4.8185	86.636	2.737	86.429	30.668	7.8565	7.756	4.9745
**75%**	48.845	6.312	5.3995	87.12	2.754	86.608	33.557	9.0262	9.4522	5.9402
**max**	54.278	6.483	6.234	90.795	2.79	87.065	47.79	11.506	50.492	6.983

## 3 Results and discussions

### 3.1 The transfer performance evaluation of the reservoir parameter prediction

After data processing, the parameters of the transfer prediction model should be determined, including the number of layers in the neural network, the number of units, the learning rate, and the optimization function. The number of LSTM hidden layers was pre-set as {2, 3, 4, 5, 6}, the learning rate as {0.1, 0.01, 0.001}, the number of neurons set as {16, 32, 64, 128, 256} and the dropout rate as {0, 0.1, 0.2, 0.3, 0.4, 0.5}. These parameters were optimized by grid search algorithm. The final parameter settings are shown in Table 6.

**Table 6:** Parameter configuration of the neural network structure.

Parameters	setting
LSTM hidden layers, units	3,(128,128,128)
Output layer	Fully connected layer
Activation function	Relu
Learning rate	0.01
Dropout rate	0
Epoch of pre-train	100
Epoch of fine-tune	100
Batch size of the source model	128
Batch size of the target model	4

To verify model robustness, four prediction models were established in this article for each transfer task with reference to Xi [44] for better comparative analysis of transfer learning:

- Model A: train the LSTM prediction model on the source well
- Model B: train the LSTM prediction model on the target well
- Model C: train LSTM prediction model after mixing source and target data
- Model D: pre-train model on the source well and finetune on the target well.

**Fig 4:**
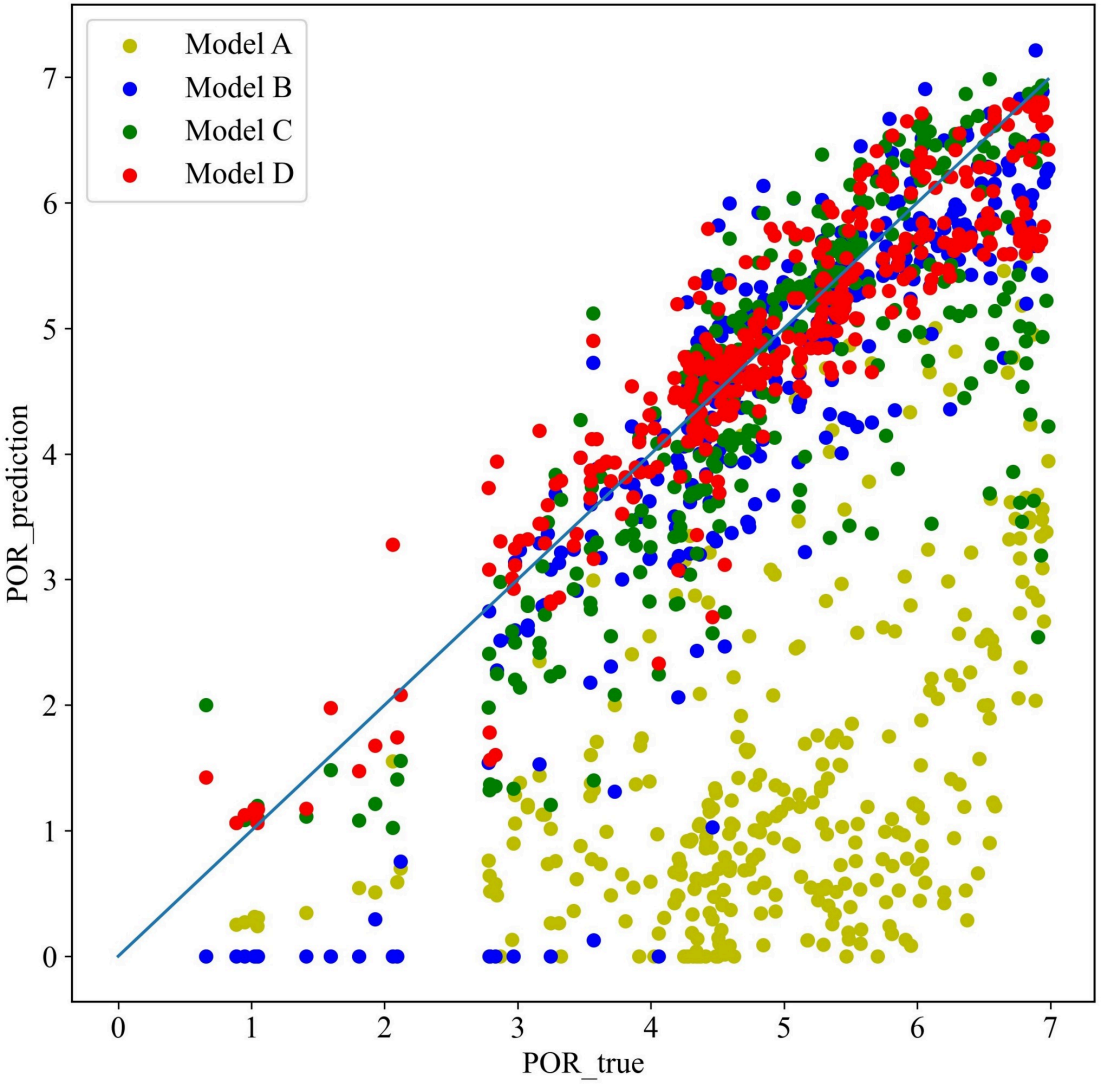
Fitting distribution of predicted and true values of the four models.

As shown in Fig 4, by comparing the fitted distributions of the four models on the predicted and true values, the prediction results using the pre-trained model (Model D) were the closest to the true values. Then, sorted by fit in descending order, the model trained using the target dataset (Model B) ranked the first, followed by the model trained directly with the mixed data (Model C), and finally the model trained directly with the source dataset (Model A).

Because Model D could extract most of the features of the source and target datasets with transfer learning, which effectively strengthened the predictive power and generalization level of the model. Among the three models trained directly, the mixed data model (Model C) had better prediction ability than the other two due to its advantage in data sample size and the ability to adjust the model with the target dataset. When comparing the two models trained directly with the target dataset (Model B) and with the source dataset (Model A), the former had the dominant advantage in terms of data feature distribution given the superior data size of the latter. This result illustrated well the necessity and significance of transfer learning trained on these two datasets with different data distributions.

To better demonstrate the effectiveness of transfer learning in such tasks, the mean squared error (MSE), the mean absolute error (MAE), the mean absolute percentage error (MAPE), and the root mean square error (RMSE) were used as typical machine learning regression prediction model evaluation metrics for the prediction performance of the four models under different tasks in this study.

$$\begin{array}{c} MSE=\frac{1}{n}\sum_{i=1}^{n}{\left(y_{i}-{\hat{y}}_{i}\right)}^{2} \end{array}$$ (4)

$$\begin{array}{c} MAE=\frac{1}{n}\sum_{i=1}^{n}\left|y_{i}-{\hat{y}}_{i}\right| \end{array}$$ (5)

$$\begin{array}{c} MAPE=\frac{1}{n}\sum_{i=1}^{n}\frac{\left|y_{i}-{\hat{y}}_{i}\right|}{y_{i}} \end{array}$$ (6)

$$\begin{array}{c} RMSE=\sqrt{\frac{\sum_{i=1}^{n}(y_{i}-{\hat{y}}_{i}{)}^{2}}{n}} \end{array}$$ (7)

Where *n* is the number of samples in the test set, ${\hat{y}}_{i}$ the predicted value of POR, and *y*_*i*_ the true value.

The performance evaluation metrics of four different models are shown in Table 7. It could be seen that the performance evaluation metrics of the proposed Pretrain-finetune model (Model D) were the most superior ones, followed by the model train on the target well (Model B), that on the mixed data of the source and target wells (Model C), and that on the source well (Model A). The maximum values of these four evaluation metrics were observed in Model A supposedly due to the huge difference in the distribution of the source and target well data. The model trained on the source well data was completely inadequate for the target well prediction task, the predictions of which were more like randomly generated values. The proposed model (Model D), compared with other three models, generated the smallest evaluation metrics because it utilized the massive dataset of source wells effectively and solved the distribution difference between the two datasets by transfer learning.

**Table 7:** Prediction performance metrics of four different models.

Model	MAE	MSE	MAPE	RMSE
A	3.4622	13.7187	0.7146	3.7038
B	0.6207	0.7725	0.162	0.8789
C	0.6492	0.8798	0.1411	0.938
D	**0.4008**	**0.2688**	**0.0893**	**0.5184**

**Fig 5:**
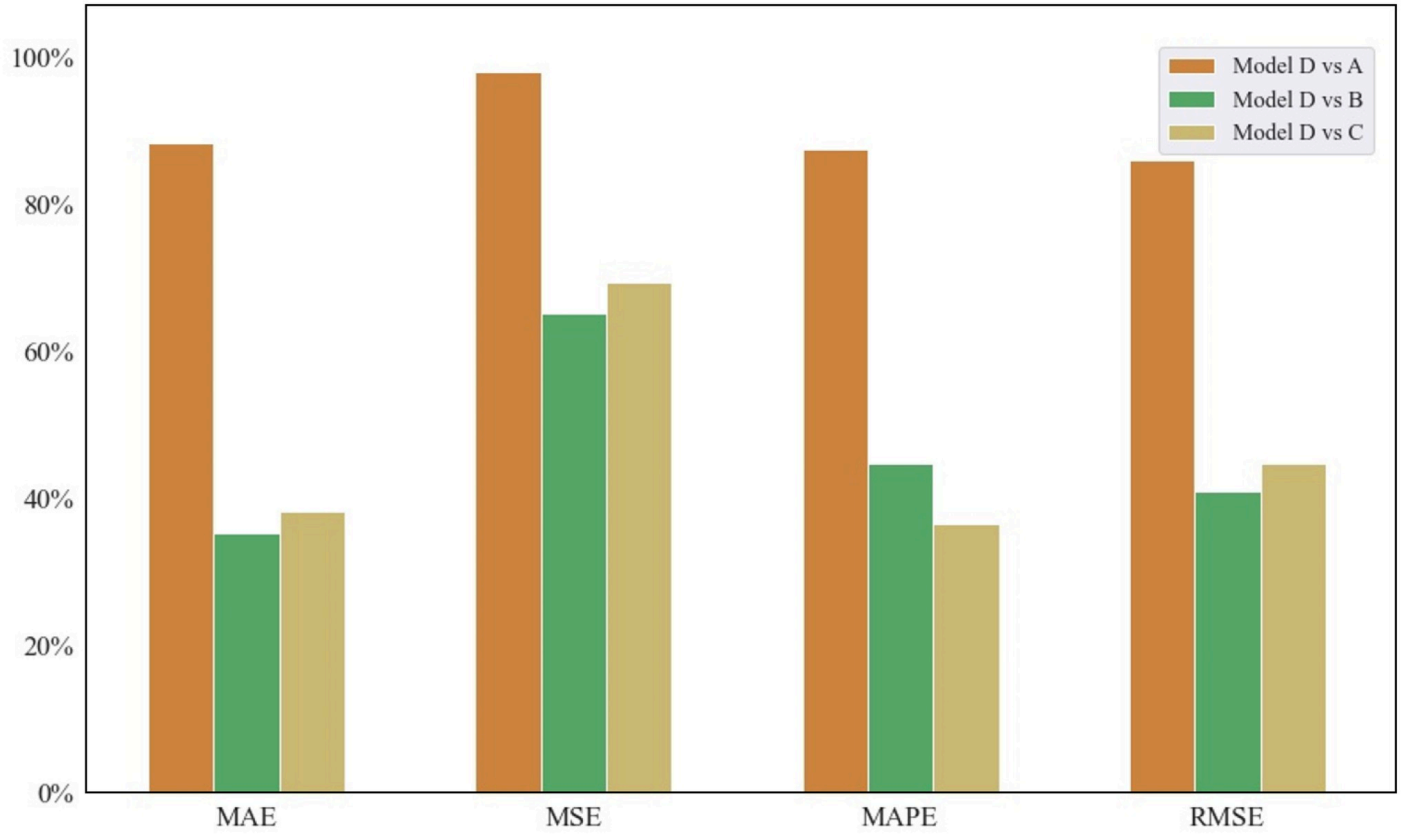
Prediction performance promotion percentages between the proposed model and other models.

Fig 5 shows the significant improvement of each metric for the prediction performance of the proposed model compared with other three models. The improvements of MAE, MSE, MAPE, and RMSE values were over 30%. Among them, the improvement of MSE value was the most evident, almost.e., approximately 100% compared with that of model A. These results showed that the Pretrain-finetune model was more competent to predict well parameters in the absence of training samples.

**Fig 6:**
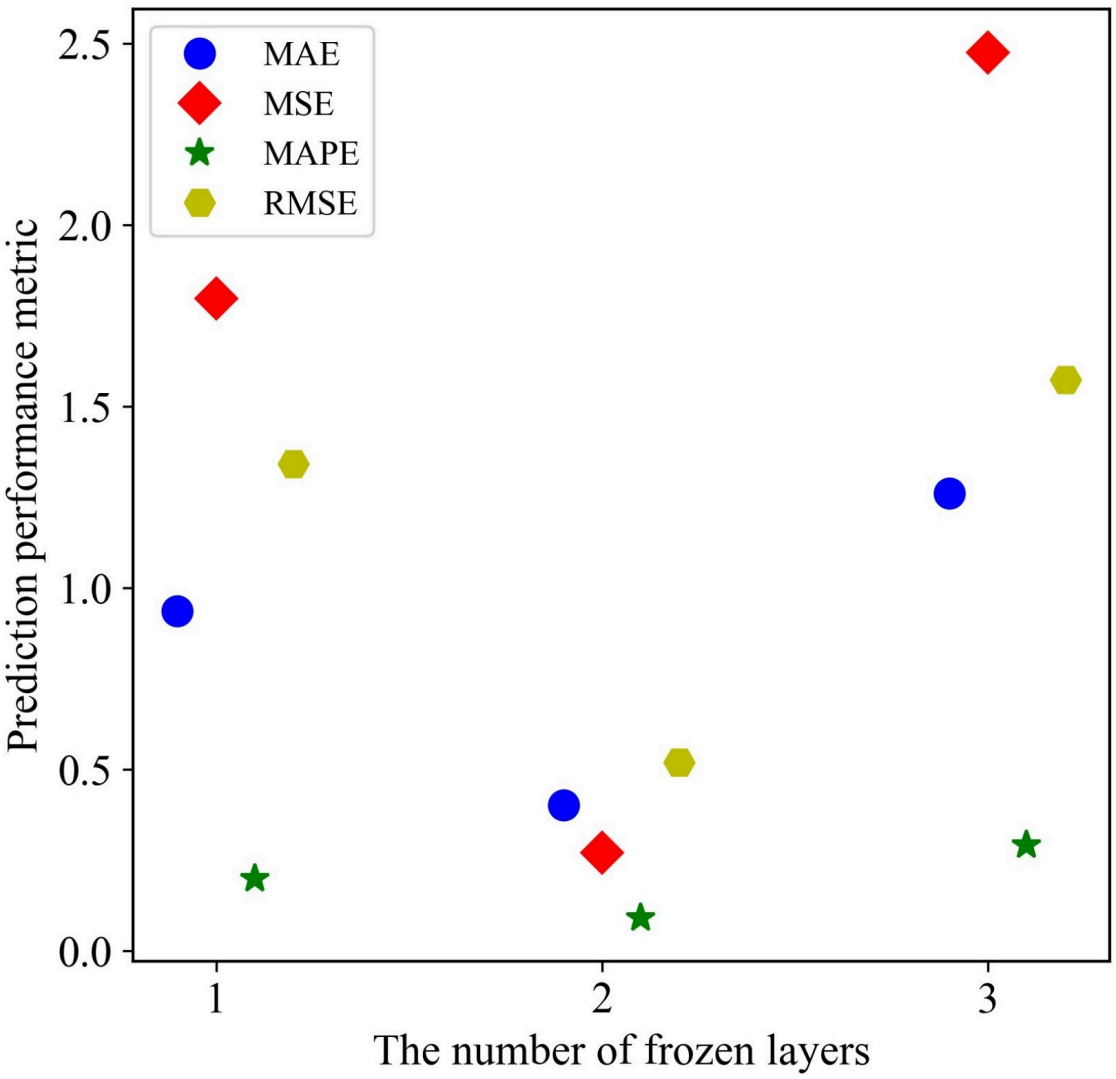
Impact of the number of frozen layers on prediction performance of the proposed model.

Another key issue is whether feature transfer is all valid. Therefore, we set up experiments on the number of frozen layers and transfer effects with reference to Jason [45]. Then, the prediction performance of the proposed model was further tested under freezing different layers, with the results shown in Fig 6. It could be seen that the model delivered best performance on the test dataset when the first two layers of the LSTM network structure were frozen, and the number of frozen layers had a significant impact on the prediction accuracy, which may be due to the fact that the proposed model needs to balance between the extracted effective knowledge and the noisy knowledge. The increase in the number of frozen layers was associated with more comprehensive retention of the effective knowledge extracted from the source dataset, and more noise knowledge that distinguished the target well. Therefore, fine-tuning the two layers at the end of the neural network could not only prevent the extracted knowledge from being biased toward the source well but also simultaneously achieved as much valid knowledge retention as possible.

### 3.2 Influence from the size of the target dataset

**Fig 7:**
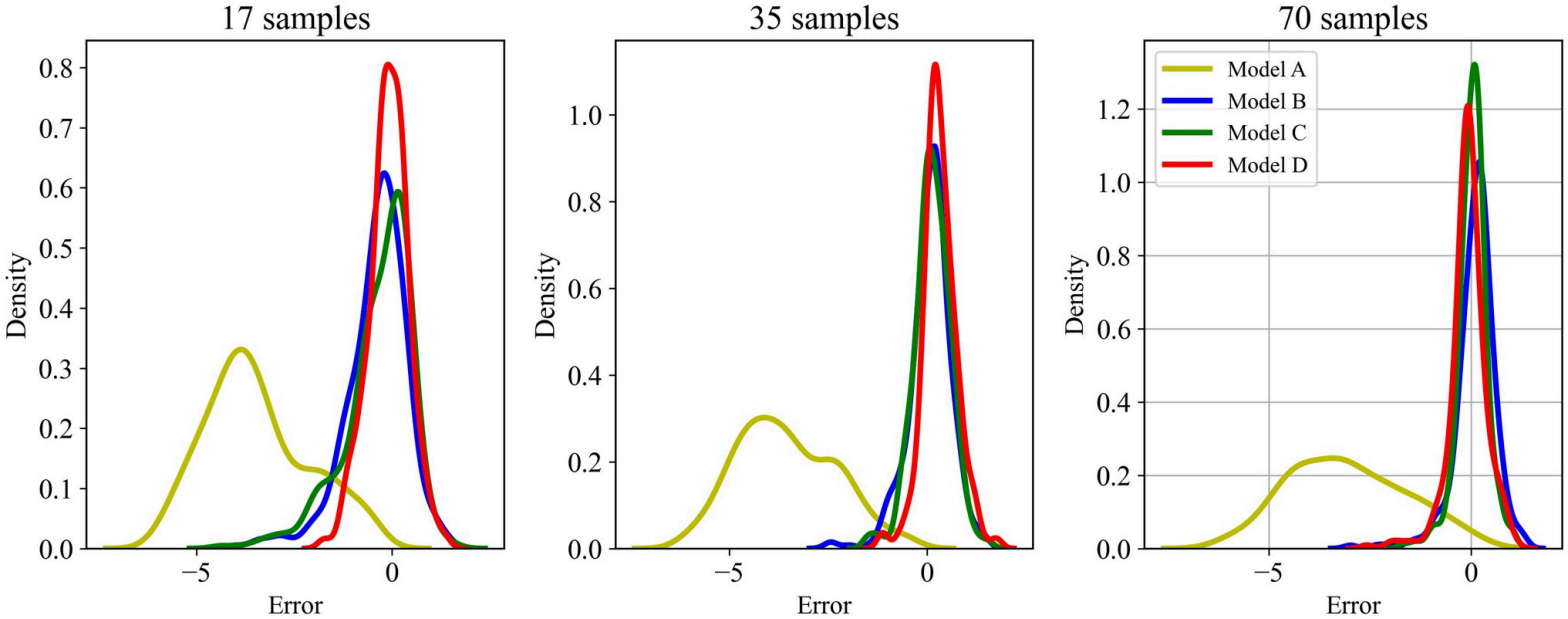
Prediction error distributions of four models with different training sample sizes.

An important problem in transfer learning is when to use transfer learning and how to avoid negative transfer. To explore this problem, target datasets of different sizes were also used in the training. The error distributions of the four models with different training sample sizes are shown in Fig 7. Higher crest of the curve makes it closer to the zero value, indicating smaller distance between the predicted and true values, and better performance of the model.

When the target dataset involved in training was small, such as 17 and 35 samples, the improvement of transfer learning was quite significant. When there were 70 samples in the target dataset, transfer learning even led to a negative transfer effect, with a prediction error larger than that of the model using the mixed data directly. It could be therefore concluded that using transfer learning could significantly improve the prediction accuracy when the target dataset involved in training had around 35 samples. However, when the number of data was close to or larger than 70, the effect of transfer learning on prediction accuracy was not significant or even counterproductive.

### 3.3 Reliability test on other target wells

To test the reliability of the proposed method, we used four models trained and predicted on two new target wells in the Chuanxi block. Gaomo 001 is still selected as the source well for transfer learning on Chuanxi 002 and 003, respectively. All parameter settings of the models remained consistent with those in Subsection 3.1, except for the different data sources. The prediction performance is shown in Table 8. It shows the same trend of forecast performance as on Chuanxi 001. The prediction accuracy is ranked from good to bad for Models D, C, B, and A, respectively. The proposed transfer learning (Model D) has an improvement of more than 30% in four evaluation metrics, which indicated that the proposed model could effectively improve the reduced prediction accuracy of most wells in the Chuanxi block due to lack of training data.

**Table 8:** Prediction performance metrics of four different models in other target wells.

Source well	Target well	Model	MAE	MSE	MAPE	RMSE
Gaomo 001	Chuanxi 002	A	3.53	13.246	0.9997	3.6395
		B	0.3397	0.1823	0.1086	0.427
		C	0.3304	0.1894	0.1025	0.4352
		D	**0.2064**	**0.0734**	**0.0715**	**0.271**
	Chuanxi 003	A	2.5627	7.0714	0.8684	2.6592
		B	0.4978	0.9214	0.1872	0.9599
		C	0.5661	0.5671	0.1887	0.753
		D	**0.3362**	**0.4795**	**0.1131**	**0.6924**

### 3.4 The transfer performance comparison of different transfer learning models

To further test the performance of the proposed method, it was compared with two other transfer learning methods. LSTM was used as the regression predictor for DANN and support vector machine regression(SVR) [46] as the regression predictor for Tradaboost. The results of comparison are shown in Table 9. Taking Chuanxi 001 well as an example, the Pretrain-finetune method was significantly better than other algorithms in all metrics. It was also found that DANN used a domain classifier trained against a feature extractor to achieve feature alignment, but it was designed primarily to solve the classification problem with its performance more dependent on the selection of the model structure parameters, thus it delivered the worst performance in this test. TrAdaboost-SVR exhibits the second best prediction accuracy, thanks to its strong source domain sample screening capability. However, due to the need to continuously calculate and update the weights of each sample, the disadvantages of large computational effort and long computation time are obvious. The proposed Pretrain-finetune method generated the highest prediction accuracy compared with the feature-based and sample-based transfer learning methods, demonstrating that this method is the best choice for this research problem.

**Table 9:** Prediction performance metrics of different algorithms.

Source well	Target well	Model	MAE	MSE	MAPE	RMSE
Gaomo 001	Chuanxi 001	TrAdaboost-SVR	0.5374	0.4715	0.3466	0.6866
		LSTM-DANN	0.7852	0.9675	0.1625	0.9836
		Pretrain-finetune	**0.4008**	**0.2688**	**0.0893**	**0.5184**

### 3.5 Overall comparison

We present a comparative analysis of our proposed TL-LSTM model alongside several established methodologies, encompassing traditional integrated learning techniques, virtual data generation-based approaches, and recurrent neural network-based transfer learning methodologies. The baseline details for each method are as follows:

- **ELM** (Ensemble Learning Machine): ELM [14] employs a range of machine learning algorithms, including Deep Neural Networks (DNN), Multiple Linear Regression (MLR), Random Forest (RF), Gradient Boosting Trees (GBT), and XGBoost. Prediction credibility within the integrated machine learner is computed as a weighted average of predictions generated by these diverse learners.
- **VSG** (Virtual Sample Generation): VSG [19] leverages deep limit learning machines, data trend estimation, and hyperspherical feature equations to generate initial virtual samples. Subsequently, a virtual sample screening process is conducted, culminating in the incorporation of virtual data as labeled data for model training.
- **TL-RNN** (RNN-based Transfer Learning): This approach utilizes Recurrent Neural Networks [47] (RNN) for feature extraction on the source domain. Subsequently, a limited amount of labeled data is employed to fine-tune the model for enhanced performance on the target domain.
- **TL-GRU** (GRU-based Transfer Learning): Similar to TL-RNN, TL-GRU harnesses Gated Recurrent Unit [48] (GRU) for feature extraction on the source domain. Fine-tuning on the target domain is carried out using a restricted set of labeled data.

The performance evaluation of TL-LSTM in comparison to the baseline results is presented in Table 10. Based on the obtained results, several noteworthy observations emerge:

**Table 10:** The test results of TL-LSTM and all baselines on the Chuanxi001 datasets.

Model	MAE	MSE	MAPE	RMSE
VSG	0.577	0.5224	0.1207	0.7227
ELM	1.2283	2.4724	0.3659	1.5723
TL-RNN	0.8424	1.1369	0.19	1.0662
TL-GRU	0.8714	1.1832	0.237	1.0877
TL-LSTM	**0.4008**	**0.2688**	**0.0893**	**0.5184**

First and foremost, our proposed TL-LSTM consistently outperforms the baseline models across all four evaluation metrics on the test dataset. This noteworthy performance advantage underscores the effectiveness of our proposed transfer learning approach, combined with the modeling capabilities of long and short-term memory, specifically in the context of predictive modeling for logging data under conditions characterized by limited sample sizes.

We identify three key aspects:

(1) The capacity of our model to extract salient and generalizable features from historical data that exhibit wide variations in geologic conditions. Notably, many existing approaches, such as VSG and ELM, rely primarily on modeling a small fraction of labeled data originating from the same well. Our approach, in contrast, leverages the potential of the entire dataset, which mitigates overfitting on the training set, a common challenge associated with limited data availability.
(2) In contrast to traditional RNN and GRU architectures, LSTM exhibits superior long-term memory and the ability to capture long-distance dependencies when dealing with sequential data. It adeptly addresses the issues of gradient vanishing and gradient explosion, which can be especially problematic when dealing with depth-dependent logging data. Our test results affirm the efficacy of LSTM in this regard.
(3) Among the methods considered, TL-LSTM stands out by achieving the highest level of performance. This outcome underscores the efficacy of our proposed amalgamation of transfer learning and the integration of short and long-term memory modeling. It reinforces our contention that this combination is particularly potent in enhancing predictive modeling accuracy in the context of logging data analysis under constrained sample conditions.

In summation, our results demonstrate the superiority of TL-LSTM in predictive modeling for logging data, shedding light on its potential for broader applicability in similar small-sample scenarios.

## 4 Conclusions

To conclude, this paper proposed a transfer learning-based LSTM model to improve the performance in terms of reservoir parameters prediction. This model improved the reduced prediction accuracy of traditional machine learning models in the context of data shortage from the same block well. An LSTM- based model was first trained on a source well. The first few layers of this model were then frozen and the remaining layers were fine-tuned using data from the target well. it enabled the reservoir parameter prediction model to learn knowledge from both the source and target wells and discard knowledge that differed significantly between the two datasets. A series of experiments were conducted to test the performance of the proposed transfer learning strategy. The main results are as follows:

(1) The Pretrain-finetune model could effectively improve the prediction performance of reservoir parameters compared with models trained only on the source data, target data, or mixing data from the source and target wells. The improvement of prediction effect was more than 30% compared with the other three models.
(2) The amount of data in the target well exerted a significant impact on the prediction performance of the proposed model; the larger the amount of data in the target well, the weaker the effect of improvement from transfer learning.
(3) This Pretrain-finetune model showed better prediction performance for reservoir parameters compared with the Tradaboost-SVR, VSG, ELM and LSTM-DANN models, indicating that the prediction of reservoir parameters with strong regional characteristics was more suitable with the model-based transfer learning methods.

The contribution of this study lies in that it explores the feasibility of transfer learning in improving reservoir parameter prediction performance against the backdrop of training data shortage, and it demonstrates the superiority of Pretrain-finetune models in such cases. In addition, this study significantly enhances practical applications in oil and gas exploration by providing a precise solution for reservoir parameter prediction. Our model enables accurate determination of reservoir structures, guides construction plans, and contributes to substantial reductions in energy consumption. This practical innovation empowers industry professionals to make more informed and sustainable decisions, optimizing resource utilization in the exploration and development processes. However, there are still many limitations and areas that can be improved. The process of parameter setting in neural networks involved too much manual intervention. In the future, the performance of transfer learning in reservoir parameter prediction can be further improved using efficient optimization algorithms capable of automatically searching for optimal hyperparameters. In addition, due to the large amount of historical data for reservoir parameter prediction but distinct block characteristics, perhaps multiple source transfer learning methods can be better and more generalized for real application scenarios where training data is in shortage. In this paper, only data from single well were used as source data, and the knowledge contained in the huge historical data has not been mined. Therefore, adoption of multiple source transfer learning methods while simultaneously solving the problem of data distribution adaptation is an area worthy of further study.

## Supporting information

S1 Data (XLSX)

S2 Data (XLSX)

S3 Data (XLSX)

S4 Data (XLSX)
